# Factors associated with measles resurgence in the United States in the post-elimination era

**DOI:** 10.1038/s41598-020-80214-3

**Published:** 2021-01-08

**Authors:** Christian Akem Dimala, Benjamin Momo Kadia, Miriam Aiwokeh Mbong Nji, Ndemazie Nkafu Bechem

**Affiliations:** 1 Department of Medicine, Reading Hospital, Tower Health System, West Reading, PA USA; 2 Infectious Diseases Unit, University Hospitals of Leicester NHS, Leicester, UK; 3 Health and Human Development (2HD) Research Network, Douala, Cameroon; 4 Department of Clinical Sciences, Liverpool School of Tropical Medicine, Liverpool, UK; 5 Department of Epidemiology, Rollins School of Public Health, Emory University, Atlanta, GA USA; 6 College of Pharmacy and Pharmaceutical Sciences, Florida A&M University, Tallahassee, FL USA

**Keywords:** Diseases, Health care, Medical research, Risk factors

## Abstract

There have been growing concerns of a potential re-establishment of measles transmission in the United States (US) in the years to come. This study aims to explore potential factors underlying the resurgence of measles in the US by objectively assessing the associations between annual incidence rates (AIR), case importation, vaccination status and disease outbreaks. Data on measles transmission between January 1st, 2001 and December 31st, 2019 were obtained from the national centres for disease control and prevention (CDC) surveillance databases and other published reports. Changes in incidence rates over time were assessed by binomial regression models. Of the 3874 cases of measles in the US over the study period, 3506 (90.5%, 95% CI: 89.5–91.4) occurred in US residents. The AIR per million population in US residents over this period was 0.60 (95% CI: 0.59–0.61), with an overall significant increase over time (*p* = 0.011). The median percentage of imported and vaccinated cases were 36% [17.9–46.6] and 15% [12.1–23.2] respectively. There was a significant decrease in the percentage of imported cases (*p* < 0.001) but not of vaccinated cases (*p* = 0.159) over time. There was a moderate and weak negative correlation between the AIR and the percentage of imported and vaccinated cases respectively (r = –0.59 and r = –0.27 respectively). On multiple linear regression there was a significant linear association between the AIR and the number of outbreaks (*p* = 0.003) but not with the percentage of imported cases (*p* = 0.436) and vaccinated cases (*p* = 0.692), R^2^ = 0.73. Strong negative and positive correlations were seen between the number of outbreaks and the percentage of imported cases (r = –0.61) and the of number states affected (r = 0.88) respectively. Despite the overall reduction in the percentage of imported cases of measles over the past two decades, pockets of internal transmission of the disease following importation via increasing number of outbreaks in unvaccinated subpopulations, reinforced by vaccine hesitancy, account for the sustained increase in measles incidence rates in the US. Controlling indigenous transmission through efficient vaccination coverage in at-risk subpopulations and among international US travellers, improved disease surveillance and rapid outbreak containment are essential in curbing the measles resurgence.

## Introduction

Even though measles had spread across North Africa and Europe in the sixteenth century, it was only imported to the Americas by European explorers in the 1650s^1,2^. Measles, however, became a notifiable disease in the United States (US) in 1912, with an estimated 6000 measles-related deaths on average annually in the first decade of reporting^3^. With the introduction of the measles vaccine in 1963, measles remained under control in the US up until the late 1980s and early 1990s when new outbreaks were reported. These new outbreaks are known to have been due to the low vaccine coverage in the affected cities and the densely populated urban areas, with Hispanics and Blacks as the mainly affected ethnicities^4,5^. Furthermore, the growing number of young mothers meant they mainly obtained immunity from vaccination rather than infection and so had lower antibody titres and antibodies to transfer trans-placentally to their children, who consequently had weaker immunities to resist measles^6^. Likewise, health care providers failed to administer the vaccine to eligible children^7^. However, the occurrence of measles in more than half of the vaccinated children aged 5–19 years of age, raised the suspicion of a possible vaccine failure^8^. After 1993, there was a significant drop in the number of measles cases and a noticeable change in the demographics of the affected population, switching from middle high-school and college students prior to then, towards adults due to the policy in place by all states that required school-aged children to receive 2 doses of the measles vaccine^6^. The downward trend continued all through the 1990s and in 2000, by way of a highly effective vaccination program, measles elimination was declared in the US^6,9^. The measles post-elimination era in the US had been relatively stable with low pockets of transmission and fairly stable incidence rates in the country^10^. However, In recent years there has been a resurgence of measles in the US with the annual median number of cases and outbreaks between 2009 and 2014 standing at more than double the numbers in the first decade following elimination^11^. These have resulted in growing concerns of a potential re-establishment of transmission of measles, and loss of the ‘measles elimination’ status by the US in the years to come if no appropriate health policy actions are taken^12^. There are suggestions this resurgence of measles could be due to the declining vaccine coverage as a result of vaccine hesitancy^13^ and an ever growing number of anti-vaccination movements as observed in some parts of the country^14^. Given the highly infectious nature of measles^15^, several factors could potentially favour the transmission of measles and consequent resurgence such as; population density; inter/intra-age contact; timing of the vaccination and waxing conferred immunity. This resurgence is likely multifactorial in origin, however, the contribution of various factors to this resurgence has not be ascertained and/or quantified. This study aimed to provide answers regarding possible reasons for the resurgence of measles in the US in recent years and propose solutions to help with its control by reviewing the epidemiology of measles transmission in the US over the past 19 years and exploring factors associated with this transmission. More specifically, this study had as objectives:

1. To describe the measles epidemiology in the US post-elimination (2001 to 2019).
2. To assess the association and correlation between the vaccination status, case importation, disease outbreaks and measles incidence rates over this period
3. To discuss potential reasons for the resurgence of measles in recent years in the US and propose potential solutions to the current measles crises.

## Methods

### Study design and data sources

This was an ecological study with trend analysis of all cases and outbreaks of measles in the US over the past two decades. The epidemiology of measles and the respective outbreaks between January 1st, 2001 and December 31st, 2019 were studied. This time period is often referred to as the post-elimination era. Data on measles were obtained from the National Centres for Disease Control and Prevention (CDC) surveillance databases including the National Notifiable Diseases Surveillance System (NNDSS)^16^, the Notifiable Infectious Disease and Conditions data, the Nationally Notifiable Infectious Diseases and Conditions, United States: Annual Tables^17^, the measles-related morbidity and mortality weekly reports (MMWR)^18^ and other published reports. Standard surveillance case definitions for measles used by the respective state health departments for reporting measles cases over the years were according to the respective CDC measles case definitions^16^.

### Data management and analysis

Data extracted from the data sources, when provided, included: the overall number of measles cases per year, the number of imported cases, the number of cases in US residents, the overall number and proportion of vaccinated and unvaccinated cases, the number and proportion of vaccinated and unvaccinated US resident cases, the number of outbreaks, the number of states affected, the number of deaths, the main countries of importation and the most frequent virus strains per outbreaks. The total and US residents annual incidence rates (AIR) per million were computed as; the overall number of measles cases and the number of measles cases in US residents for that year respectively, divided by the crude estimates of the USA population at risk for that year as per the US census bureau data^19^. Percentages of imported and vaccinated cases were calculated as; the number of imported and vaccinated cases respectively divided by the total measles cases and US resident cases respectively. Cases were considered as vaccinated if they had received at least a dose of the measles vaccine, while unvaccinated cases were considered as those who reported not having received the vaccine and those whose vaccination status were unknown. All statistical analyses were done using STATA 14 statistical software. Categorical variables were reported as frequencies and percentages, continuous variables were reported as means with standard deviations and medians with interquartile ranges as appropriate. Graphs were plotted for annual incidence rates, vaccinated US resident cases and percentages, imported cases and percentages over time. Binomial regression models were used to assess for changes in annual incidence rates and percentage of imported and vaccinated cases over time. Pearson’s correlation coefficient was used to assess for the linearity between continuous variables. Linear regression models were built to assess for linear associations between importation percentages, vaccination percentages, outbreaks and the AIR and corresponding regression equations were derived and reported. Multiple regression was used to assess for these linear associations while controlling for potential confounders. A complete case analysis approach was used to manage missing data as the complete cases were a random sample of the overall data. Statistical significance was considered for *p* values < 0.05.

### Ethical considerations and reporting

This was an analysis of publicly available disease surveillance data and ethical approval was therefore not required. The ‘Strengthening the Reporting of Observational studies in Epidemiology’ (STROBE) guidelines were used for the reporting of this study (Additional file 1).

## Results

### General characteristics

Between 2001 and 2019, there was a total of 3874 cases of measles in the USA, 3506 (90.5%, 95% CI: 89.5–91.4) of whom were US residents, giving a median of 85 [55–188] total and 65 [42–178] US resident cases of measles per year. A total of 160 outbreaks were reported over this period with a yearly median of 6 outbreaks and 18 states affected by the disease (Table 1). A median of 36% [17.9–46.6] of cases per year were due to international importation and a median of 15.1% [12.1–23.2] of US cases occurred in vaccinated people (Table 1). Up to a median of 66.7% of vaccine-eligible cases declined to be vaccinated due to religious beliefs (Table 1). A total of 8 deaths were reported over this period. A summary of the clinical characteristics of the measles cases for each year is presented in Table 2.

**Table 1:** Clinical characteristics of measles cases between 2001 and 2019.

Characteristics, n = 19	Numbers
**Overall cases**	
Total	3874
Median/year [IQR]	86 [55–188]
**US resident cases**	
Total	3506
Median/year [IQR]	65 [42–178]
**Imported cases**	
Total	734
Median/year [IQR]	27 [21–54]
**Imported cases percentage **(**%**)	
Median/year [IQR]	36 [17.9–46.6]
**US residents vaccinated cases, n = 16**	
Median/year [IQR]	10 [7–27]
**US residents vaccinated percentage **(**%**)**, n = 16**	
Median/year [IQR]	15.1% [12.1–23.2]
**Outbreaks**	
Total	160
Median/year [IQR]	6 [4–10]
**States affected**	
Median/year [IQR]	18 [16–23]
**Deaths**	
Total	8
**Percentage of vaccine refusal due to religious beliefs **(**%**)**, n = 6**	
Median/year [IQR]	66.7% [44–67]
Range	36—76

**Table 2:** Annual measles incidence rates, percentage of imported and vaccinated cases per year in the United States, 2001–2019.

Year	Total cases	US resident cases	US AIR/million (95% CI)	Imported cases (%)	Vaccinated cases (%)	Out- breaks	States affected	Deaths
2001	116	81	0.28 (0.23–0.34)	54 (46.6)	24 (29.6)	10	22	0
2002	44	36	0.13 (0.09–0.16)	18 (40.9	5 (13.9)	3	17	0
2003	56	38	0.13 (0.09–017)	24 (42.9)	9 (23.7)	3	15	2
2004	37	24	0.08 (0.05–0.11)	27 (73.0)	4 (16.7)	2	13	0
2005	66	59	0.20 (0.15–0.25)	24 (36.4)	7 (11.9)	3	16	0
2006	55	42	0.14 (0.10–0.18)	31 (56.4)	11 (26.2)	4	16	0
2007	43	31	0.10 (0.07–0.14)	29 (67.4)	7 (22.6)	4	15	0
2008	140	127	0.42 (0.36–0.47)	25 (17.9)	7 (5.5)	9	15	0
2009	72	65	0.21 (0.17–0.26)	21 (29.2)	8 (12.3)	8	17	2
2010	63	47	0.15 (0.11–0.19)	39 (61.9)	6 (12.8)	4	17	2
2011	222	196	0.63 (0.58–0.68)	80 (36.0)	30 (15.3)	17	31	0
2012	55	43	0.14 (0.10–0.17)	21 (38.2)	11 (25.6)	4	18	0
2013	187	164	0.52 (0.46–0.57)	51 (27.3)	13 (7.9)	10	18	0
2014	667	658	2.07 (1.93–2.21)	63 (9.4)	53 (8.1)	23	29	0
2015	188	178	0.55 (0.50–0.61)	26 (13.8)	36 (20.2)	6	23	1
2016	86	55	0.17 (0.13–0.21)	17 (20.0)	NR	4	20	0
2017	120	114	0.35 (0.30 – 0.40)	21 (17.5)	NR	7	19	0
2018	375	337	1.03 (0.93–1.14)	82 (21.9)	NR	17	25	0
2019	1282	1211	3.68 (3.51–3.85)	81 (6.3)	179 (14.8)	22	31	0

NR–Not reported.

### Trends in the measles annual incidence rates, imported cases and vaccination status

**Figure 1:**
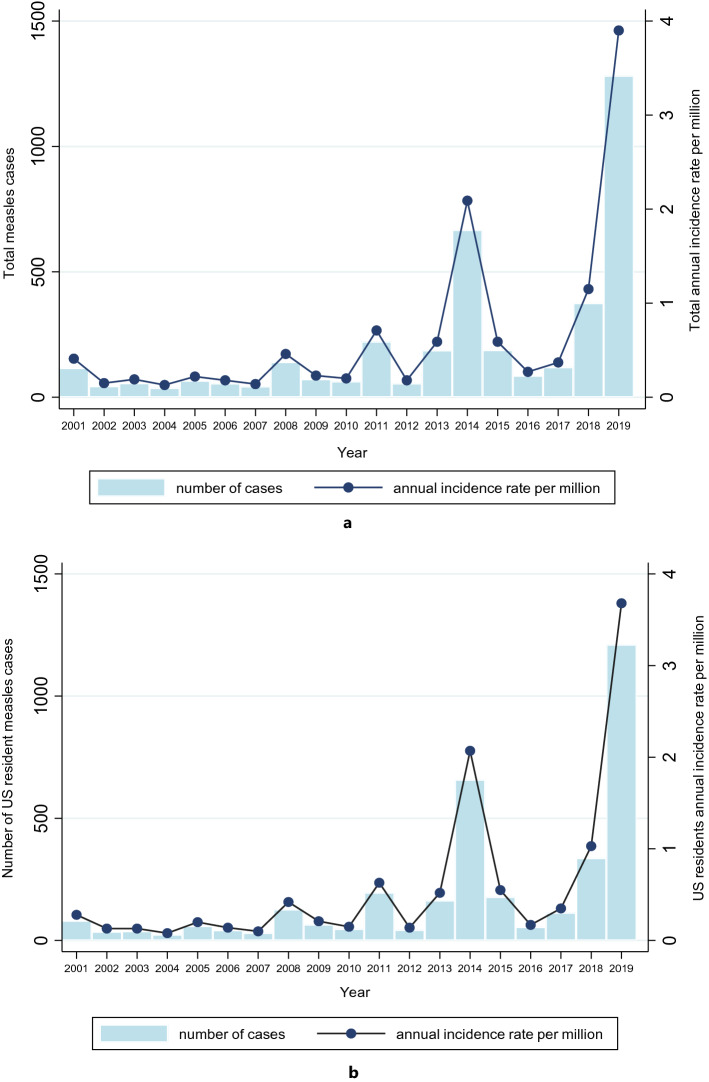
(**a**) Total annual incidence rate of measles and total number of measles cases between 2001 and 2019. (**b**) Annual incidence rate of measles and number of measles cases in US residents between 2001 and 2019. The light blue shading represents the number of cases and the connected line represents the annual incidence rate (AIR) per million population. The total AIR and the AIR in US residents was relatively constant between 2001 and 2013, but there were subsequent peaks in 2014 and 2019.

**Figure 2:**
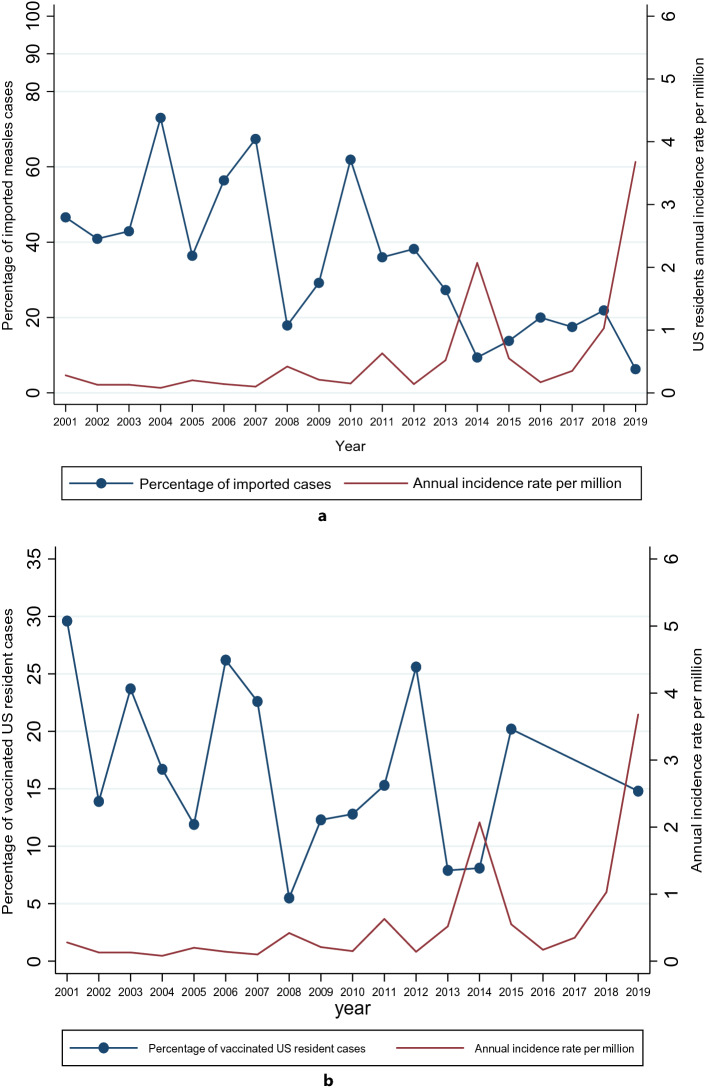
(**a**) Annual incidence rate of measles in US residents and the percentage of imported cases between 2001 and 2019. (**b**) Annual incidence rate of measles in US residents and the percentage of vaccinated cases between 2001 and 2019. The red line represents the annual incidence rate (AIR) per million population and the blue connected line represents the percentage of imported cases and vaccinated respectively. Missing data on the percentage of vaccinated US resident cases for 2016 and 2017 in Fig. 2b. There was a decrease in the percentage of imported cases from 46.6% in 2001 to 6.3% in 2019.

The overall measles incidence over this 19-year period was 0.66 (95% CI: 0.65–0.67) per million population and 0.60 (95% CI: 0.59–0.61) per million population in US residents. The annual number of measles cases in US residents ranged from 24 to 1211 cases, and the AIR per million in US residents ranged from 0.08 (95% CI: 0.05–0.11) to 3.68 (95% CI: 3.48–3.89). There was an overall increase in the AIR in US residents over time from 0.28 (95%CI: 0.23—0.34) in 2001 to 3.68 (95% CI: 3.48—3.89) in 2019, *p* = 0.011. The percentage of cases imported ranged from 6.3% to 73% and an overall decrease in the percentage of imported cases over time was observed from 46.6% in 2001 to 6.3% in 2019, *p* < 0.001. The percentage of vaccinated US residents ranged from 5.5% to 29.6% and decreased over time though not significantly from 29.6% in 2001 to 14.8% in 2019 (*p* < 0.159). Trends in the total and US residents AIRs, percentage of imported and vaccinated cases over time are presented in Figs. 1 and 2.

### Association between measles annual incidence rates, imported cases, case vaccination status and outbreaks

Table 3 summarises the respective linear regression coefficients, intercepts, p values and R^2^ values for the linear associations between the percentage of imported cases, vaccinated cases, outbreaks and the AIR in US residents.

**Table 3:** Linear regression model for annual incidence rate in US residents, percentage of imported and vaccinated cases and outbreaks.

Parameters	Regression Coefficient	Intercept	P value	R-square	Correlation Coefficient
**Annual incidence rate**					
Percentage of imported cases	– 0.027	1.503	0.008	0.35	– 0.59
Percentage of vaccinated cases	– 0.036	1.191	0.308	0.07	– 0.27
**Outbreaks**					
Percentage of imported cases	– 0.205	15.579	0.006	0.37	– 0.61
Percentage of unvaccinated cases	0.329	– 19.182	0.249	0.12	0.35
**States affected**					
Outbreaks	0.742	13.593	< 0.001	0.78	0.88

**Figure 3:**
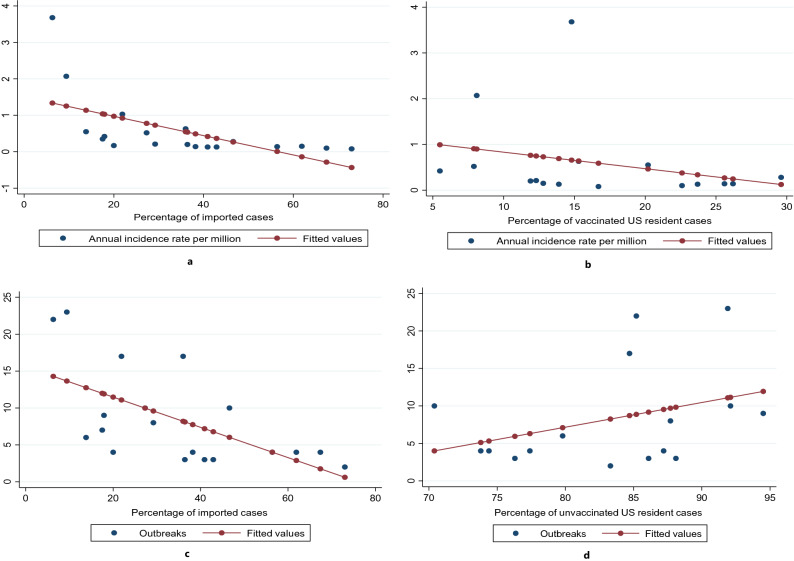
(**a**) Linear regression model of the association between the annual incidence rate and the percentage of imported cases. (**b**) Linear regression model of the association between the annual incidence rate and the percentage of vaccinated cases. (**c**) Linear regression model of the association between the number of outbreaks and the percentage of imported cases. (**d**) Linear regression model of the association between the number of outbreaks and the percentage of unvaccinated cases.

There was a moderate negative correlation and linear association between the percentage of imported cases of measles and the AIR in US residents (r = -0.59 and *p* = 0.008 respectively) (Fig. 3a and Table 3).

Regression equation: AIR = 1.503–0.027 * percentage of imported cases.

There was a weak negative correlation but no linear association between the percentage of vaccinated cases and the AIR in US residents (r = -0.27 and *p* = 0.308) (Fig. 3b and Table 3).

Regression equation: AIR = 1.191–0.036 * percentage of vaccinated cases.

There was a strong negative correlation and linear association between the percentage of imported cases and the number of outbreaks (r = -0.61 and *p* = 0.006 respectively) (Fig. 3c and Table 3).

Regression equation: Outbreaks = 15.579–0.205 * percentage of imported cases.

On the other hand, there was a weak positive correlation and no significant linear association between the percentage of unvaccinated cases and the number of outbreaks (r = 0.35 and *p* = 0.249 respectively) (Fig. 3d and Table 3).

Regression equation: Outbreaks = -19.182 + 0.329 * Percentage of unvaccinated cases.

**Figure 4:**
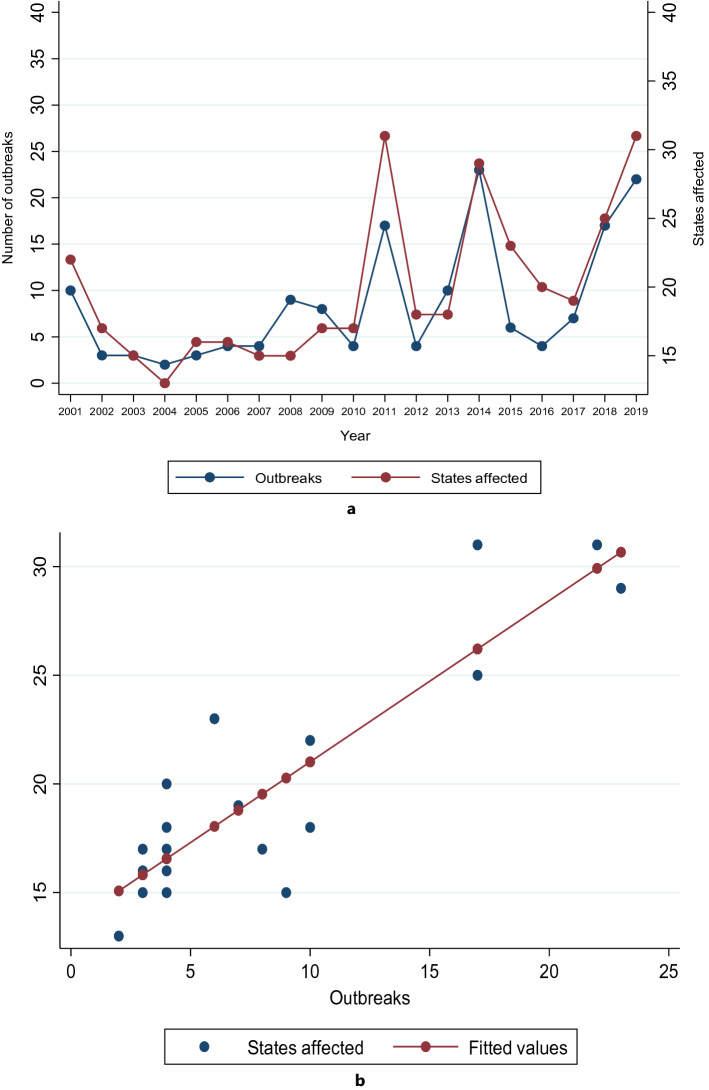
(**a**) Number of measles outbreaks and number of states affected by measles between 2001 and 2019. The red connected line represents the number of states affected and the blue connected line represents the number of outbreaks. There was a significant increase in the number of outbreaks from 10 in 2001 to 22 in 2019. There was a significant increase in the number of states affected from 16 in 22 in 2001 to 31 in 2019. (**b**) Linear regression model of the association between the number of outbreaks and the number of states affected.

There were significant increases in the number of outbreaks (*p* = 0.002) and states affected (*p* = 0.002) between 2001 and 2019 (Fig. 4a). There was a strong positive correlation and linear association between the number of outbreaks and the number of states affected by measles (r = 0.88 and *p* < 0.001 respectively) (Fig. 4b and Table 3).

Regression equation: States affected = 13.593 + 0.742 * outbreaks.

On multiple regression, there was a significant linear association between the AIR in US residents and the number of outbreaks (*p* = 0.003) and no more with the percentage of imported cases (*p* = 0.436) after adjusting for number of outbreaks and vaccination status.

Multiple regression equation: AIR = -0.123–0.008 * percentage of imported cases + 0.009 * percentage of vaccinated cases + 0.106 * outbreaks. (R^2^ = 0.73, *p* = 0.436, *p* = 0.692, and *p* = 0.003 respectively).

## Discussion

This study sought to review the epidemiology of measles in the US over the past two decades and assess for potential associations between the percentage of imported cases, vaccinated cases, outbreaks and the annual incidence rates. There has been an overall significant increase in the incidence rate of measles in US residents, the number of outbreaks and states affected, and a decrease in the percentage of imported cases, over time. Despite this increase in measles incidence in the US over time, in absolutely terms this incidence rate remains low, and relatively small when compared to the incidence of measles worldwide^20^.

It is believed that since the elimination of measles in the USA, most measles cases and transmission in the US have been from importation due to international travel^21^. The large outbreaks in several countries in Europe in recent years^20^ have significantly reinforced the disease burden due to importation by non-vaccinated international US travellers. Across the Atlantic for example, the United Kingdom which initially achieved measles elimination in 2017, lost this status of elimination in 2018 due to a rapid rise in the number of measles cases and outbreaks across the country^22^. Nevertheless, an overall significant reduction in the percentage of imported cases has been observed over the past two decades. This is in part due to the call for accelerated action in 2015 among member states of the World Health Organisation (WHO) Europe region to gear up against the elimination of measles and rubella^23^**.** This has helped to curb the number of imported cases from Europe through improved disease surveillance, vaccination/immunization strengthening, outbreak preparedness and response. Even with complete control of indigenous transmission of the disease, controlling measles importation from Europe and Asia will remain a challenge^24^. Much more therefore needs to be done with regards to the vaccination of international US travellers. As reported in a study by Hyle et al., high proportions of US vaccine-eligible children (44.1% of measles-mumps-rubella (MMR) vaccination–eligible infants, 56.5% of MMR vaccine–eligible preschool-aged travellers, and 88.5% of MMR vaccine–eligible school-aged travellers) go unvaccinated, with the principal reasons for non-vaccination being; guardian refusal and clinician decision in similar proportions^25^. In up to 75% of cases, failure to identify MMR-eligible individuals was found to be the reason for clinician refusal to administer the vaccine^25^. This highlights the often-overlooked role of clinicians in ensuring adequate vaccine coverage. Likewise among the guardian refusal reasons, in up to 75% of cases, the guardians were not concerned about the illness, and less than 10% expressed concerns about vaccine safety^25^. Clinician involvement through ways of adequate education on current vaccination guidelines and their subsequent education of guardians through pre-travel advice in travel clinics and beyond, could be essential in addressing measles cases imported by non-vaccinated international US travellers. A stricter MMR vaccination policy depending on travel destination among international US travellers could help with curbing disease importation and transmission. Likewise, compulsory measles vaccination policies could be extended to people working in settings with populations that have international travellers.

It is, however, worth noting that with the decreasing percentage of imported cases of measles in the US over the years, the increasing number of outbreaks across the country could suggest that internal transmission of the disease following importation, most probably among unvaccinated communities, could be driving the sustained increase in the AIR of measles in the US. Failure to vaccinate is traditionally known to be the main reason for indigenous transmission of the disease following importation. As reported by previous studies, measles outbreaks occurred largely in communities with high proportions of unvaccinated people, with restrictions on vaccination^9^. High vaccination coverage with effective vaccines is therefore key in controlling measles transmission in the US^26^. Sustained disease transmission in a population with a high vaccination coverage should also raise the concern of a potential waxing conferred immunity or vaccine failure among vaccinated subjects. The study by Clemmons et al., is rather in favour of failure to vaccinate rather than failure of the vaccine as the main reason for persistent disease transmission between 2001 and 2015^10^.

The increasing number of outbreaks and states affected by measles over the years implies continued surveillance of pockets of transmissions and rapid containment of local outbreaks are essential to prevent the spread of the disease to other states and the subsequent re-establishment of disease transmission post-elimination. Effective local containment can be achieved by ensuring response teams are set-up in advance to expedite containment. Extra proposed measures could involve actively searching for additional cases, administering the vaccine to every one above 6 months of age in addition to susceptible contacts, and introducing social distancing measures in extreme cases^27^.

Another important finding was the high percentage of vaccine-eligible individuals who declined vaccination due to religious beliefs. Currently, the measles vaccine, which is a live attenuated vaccine, is administered as part of the MMR or the measles-mumps-rubella-varicella (MMRV) combination together with the mumps, rubella and varicella vaccines respectively. The CDC recommends it is administered to all children 12 months of age and older, susceptible adolescents and adults without documented evidence of immunity^6^. The first dose is often given on or after the first birthday and the second dose at least 4 weeks after, and routinely administered at age 4–6 years. Despite these recommendations, vaccine hesitancy has been gaining grounds within the USA with reported instances in states like Texas^14^. Vaccine hesitancy negatively affects vaccine coverage and hence the herd immunity conferred unto non-immune individuals. Vaccine hesitancy is of public health concern as current data suggest that just a 5% drop in MMR coverage leads to a threefold increase in measles (age group 2–11 years) cases yearly and an equivalent $2.1 million cost on the public sector, constituting a significant economic burden to the US^28^. As a highly infectious disease, still endemic in several regions in the world, measles transmission is expected to persist and the identification of cases among travellers entering the US will always constitute a challenge. Therefore, controlling indigenous transmission by taking a step further on the current control measures is essential in decreasing the burden of measles on the US population.

The interpretation of our findings should take into consideration the following limitations: As with all ecological studies, there is the possibility of the ecological fallacy when inferences are made at individual level. This study analysed population data over the past two decades, and our findings on vaccination status and case importations may therefore not apply to individuals in specific communities. Despite the use of standard case definitions, variations in case detection and consequently annual incidence rates are highly dependent on the sensitivity of the surveillance systems which tends to vary over time. The findings of this study may not apply to other settings with similar or different measles epidemiology. Despite these limitations, this study provides potential reasons for the resurgence of measles in the US in recent years and possible solutions to help with its control.

## Conclusions

Despite the overall reduction in the percentage of imported cases of measles in the US over the past two decades, pockets of internal transmission of the disease following importation via increasing number of outbreaks in unvaccinated subpopulations, reinforced by vaccine hesitancy, account for the increasing incidence rates of the disease in the US. Taking a step further on the current control measures to control indigenous transmission through efficient vaccination coverage in at-risk subpopulations and among international US travellers, improved disease surveillance and rapid outbreaks containment are essential in curbing the resurgence of measles in the US.

## Data Availability

The datasets used and/or analysed during the current study are available from the corresponding author on reasonable request.

## Abbreviations

AIR: Annual incidence rate
CDC: Centres for disease control and prevention
IQR: Interquartile range
MMR: Measles-Mumps-Rubella
MMRV: Measles-Mumps-Rubella-Varicella
MMWR: Measles-related morbidity and mortality weekly reports
NNDSS: National notifiable diseases surveillance system
SD: Standard deviation
STROBE: Strengthening the reporting of observational studies in epidemiology
US: United states
WHO: World health organisation
